# Protocatechuic acid: A novel detoxication agent of fumonisin B1 for poultry industry

**DOI:** 10.3389/fvets.2022.923238

**Published:** 2022-07-26

**Authors:** Fei Wang, Yi Chen, Huilong Hu, Xinyi Liu, Yihui Wang, Muhammad Kashif Saleemi, Cheng He, Md Atiqul Haque

**Affiliations:** ^1^Key Lab of Animal Epidemiology and Zoonoses of Ministry of Agriculture and Rural Affairs, College of Veterinary Medicine, China Agricultural University, Beijing, China; ^2^Department of Pathology, University of Agriculture, Faisalabad, Pakistan; ^3^Department of Microbiology, Faculty of Veterinary and Animal Science, Hajee Mohammad Danesh Science and Technology University, Dinajpur, Bangladesh

**Keywords:** fumonisin B1, protocatechuic acid, D-glucose, silymarin, detoxification, chickens

## Abstract

Fumonisin B1 (FB1) is a major fusarium mycotoxin that largely contaminates feedstuffs and foods, posing a health risk to both animals and humans. This mycotoxin can enter the human body directly through contaminated food consumption or indirectly by toxins and their metabolites. In a prior study, feed-borne FB1 is one of the leading mycotoxins in breeder eggs, leading to reduced hatchability and gizzard ulceration in chicken progenies. Currently, no effective way is available to remove FB1 from feeds and human-contaminated foods. We hypothesize that FB1 can be reduced to low risk by protocatechuic acid (PCA). To assess the ability of FB1 to be degraded *in vivo*, 1 ppm of FB1 was treated with PCA, or D-glucose, or silymarin, or anti-FB1 monoclonal antibody. Our study revealed that both D-glucose and PCA exhibited 53.4 and 71.43% degradation, respectively, at 80°C for 2 h, while 35.15% of FB1 detoxification was determined in the silymarin group at 60°C for 0.5 h. A dose-dependent manner was found after treatment with D-glucose or PCA at 80°C for 2 h. As for detoxification of anti-FB1 monoclonal antibody, the 1:3,000 dilution induced significant FB1 detoxification, accounting for 25.9% degradation at 25°C for 2 h. Furthermore, 50 SPF 11-day-old embryonated eggs were divided into 10 groups, with five eggs per group. Post treatment with PCA or D-glucose, or silymarin or anti-FB1 monoclonal antibody, the treated samples were inoculated into albumens and monitored daily until the hatching day. Consequently, 100% of the chickens survived in the D-glucose group and other control groups, except for the FB1 control group, while 80, 80, and 60% hatching rates were found in the PCA-treated group, the anti-FB1 monoclonal antibody-treated group, and the silymarin-treated group. Additionally, both the FB1 group and the silymarin-treated group yielded lower embryo growth than other groups did. Postmortem, lower gizzard ulceration index was determined in the PCA-treated group and the anti-FB1 monoclonal antibody-treated group compared to those of the silymarin-treated group and D-glucose-treated group. Based on the above evidence, PCA is a promising detoxification to reduce FB1 contamination in the poultry industry, contributing to the eradication of mycotoxin residuals in the food chain and maintaining food security for human beings.

## Introduction

Fumonisins (FBs) are nonfluorescent, water-soluble, and heat-stable mycotoxins produced by *Fusarium* (*verticillioides, proliferatum, anthophilum, dlamini, fujikuroi, globosum, napiforme, nyagamai, oxysporum*) and *Aspergillus* (*awamori, niger*) fungi. They primarily contaminate corn (maize), corn-derived products, asparagus, sorghum, beer, rice, soybeans, and animal feed, as well as the food chain, threatening to food safety and minimizing animal production (1–4). According to recent estimates, 60–80% of mycotoxin contamination occurs naturally in food and feed, posing a risk to human and animal health worldwide (5). Mycotoxins can enter the human body directly through the consumption of plant-based foods with primary contamination or indirectly through the transfer of toxins and their metabolites into animal-derived products (milk, meat and eggs) with secondary contamination. They can also enter every stage of the food chain and food handling *via* a contaminated source (1, 6). The B-series of FBs are the most common contamination, with FB1 being the most prevalent (70–80%) followed by FB2 and FB3, causing neurotoxicity, hepatotoxicity, nephrotoxicity, immunotoxicity, and embryotoxicity in both animals and humans (2, 3, 5). FB1 induces atherosclerosis in monkeys, porcine pulmonary edema (PPE) syndrome and hydrothorax in pigs, hepatic and renal damage and immunosuppression in poultry, equine leuko-encephalomalacia (ELEM) in horses, and kidney and renal cancer in rodents, as well as primary liver cancer, esophageal cancer, neural tube defects (NTDs), and idiopathic congestive cardiopathy (ICC) in humans (7–9). A recent report showed that FB1 caused growth retardation, developmental abnormalities, neural tube defects in Brown Tsaiya Duck embryos, and negative structural alterations in bone tissues in young chickens, which can affect bone mechanics and increase the risk of bone fractures (10). Regarding harmful consequences, Codex Alimentarius Commission has defined a maximum tolerable limit (MTL) for the total of FB1 and FB2 in unprocessed maize of 4,000 and 2,000 μg/kg for maize and maize-based products, respectively (7, 11). For cereal-based items, the European Union (EU) and Food and Drug Administration (FDA) in the US set FB1 and FB2 limits of 800–4,000 and 2,000–4,000 μg/kg, respectively (1, 12, 13). From 2016 to 2018, FB1 was identified in 57.1% maize samples from eight Chinese provinces, with an average concentration of 1.9 μg/g. The maximum FB levels have been reported in staple foods/cereals during 2015–2020, 6th China Total Diet Study (TDS), with estimated daily intakes (EDIs) of 104.9 ng/kg of body weight/day and 5.25% of the provisional maximum tolerable daily intake (PMTDI) for the general population, while double exposure to consumers was identified (14). Based on the species and the animal ages, the EU recommended values for FB1 and FB2 are 60,000 μg/kg for maize and maize products in feed materials and 5,000–50,000 μg/kg for supplemental and complete feedstuffs. The FB-contaminated feed has been recorded as greater than regulatory control in several nations across the world (15, 16).

Aflatoxin B1 (AFB1) in combination with FB1 can increase reactive oxygen species (ROS) and promotes oxidative stress in mice, resulting in protein, lipid, and DNA oxidation, as well as toxicity (16). Due to synergistic or additive actions, co-contamination of mycotoxins can result in toxicological interactions and act *via* multiple stages of the same toxic pathway or when one mycotoxin absorbs another, rendering some mycotoxins dangerous even at low concentrations (15). In a prior study, we discovered that residual FB1 contamination, both alone and in combination with DON, is linked to low hatchability and gizzard ulcerations in chicken progenies, jeopardizing the sustainable poultry industry (6).

Mycotoxin binders, also known as adsorbents or sequestering agents, have been used to detoxify animal feed by binding mycotoxin and blocking its absorption in the gastrointestinal tract, where the delimited toxins can be removed through animal feces or urine (1). Inorganic binders, such as hydrated sodium calcium aluminosilicate (HSCAS), bentonite, montmorillonite, and zeolite, have been demonstrated to reduce the toxicity of AFs and DON in poultry. Bentonite clays were found to protect chickens against the cumulative toxicities of AFs + FBs and AFs + OTA in experimental trials, but the clays did not entirely alleviate the toxic effects of AFs or FBs (17). The addition of activated charcoal, bentonite, and fuller's earth to AF-contaminated feed improved the body weight of broilers by 63–100%; however, the random binding of activated charcoal with nutrients in feed limited its usage in acute cases where severe toxicosis or death was inevitable (1). Nonetheless, one of the significant drawbacks of these adsorbents is that they can bind vitamins, microelements, and macroelements, as well as other vital elements, lowering the nutritional content of the feed (17). Organic binders, such as microbial cell walls, peptidoglycans, chitosan, chitin, and enzymes, are recommended as feed additives due to their health and environmental benefits (17). Cell wall components comprising beta-D-glucans and glucomannans of yeast (*Saccharomyces cerevisiae* and *Candida utilis*) can readily bind with AFB1 (90%), FBs (72%), ZEN, OTA (55%), and T-2 toxin (1, 17). A purified extracellular enzyme from the bacterium *Myxococcus fulvus*, myxobacteria aflatoxin degradation enzyme (MADE), effectively decreased AFG1 (96.96%) and AFM1 (95.80%), while OTA was greatly reduced (74.8–84.9%) by a carboxypeptidase and peptides present in the liquid cultures of *Bacillus subtilis* (1). According to a recent study, the hydrolase and transferase enzymes of *Serratia marcescens* have shown the FB1 degradation (37%) efficiency. However, microbial methods may decrease the food quality by absorbing nutrients and emitting byproducts into the food matrices, and they are also more costly (18). These latter studies highlight the importance of careful characterization and evaluation of mycotoxin binders for use in specific mycotoxin contamination as well as appropriate *in vivo* models of target animal species to ensure efficacy and safety.

Protocatechuic acid (3,4-dihydroxybenzoic acid, PCA) is a naturally occurring phenolic acid found in a variety of edible plants, vegetables, fruits, spices, nuts, rice, crops, legumes, plant-derived beverages, and herbal medicines having diverse biological activities (19, 20). *In vivo* and *in vitro* studies revealed that PCA has anti-inflammatory, antioxidant, antibacterial, antiviral, anticancer, antiaging, antidiabetic, antitumoral, anti-asthma, antiulcer, antispasmodic, antifibrotic, analgesic, cardiac, hepatoprotective, neurological, and nephro-protective properties (21). PCA was discovered to be effective against *avian influenza virus* (AIV) and *infectious bursal disease* (IBD) virus in our pioneer investigation, and it may improve chicken health by improving both the humoral and cellular immune responses (22–24). PCA showed increased antimicrobial potential against Gram-positive (e.g., *Staphylococcus aureus, Streptococcus iniae, Bacillus cereus, and Listeria monocytogenes*), Gram-negative (e.g., *Escherichia coli, Proteus mirabilis, Pseudomonas aeruginosa, Klebsilla pneumoniae, Acinetobacter baumannii, Salmonella typhimurium, Helicobacter pylori*, and *Yersinia enterocolitica*) bacteria, and fungi (e.g., *Candida albicans* and *Microsporum audouinii*) when used alone or in combination with other antibiotics, so it could be a prospective use for contamination prevention and food conservation (25–29). However, the effect of PCA detoxification on FB1 has not been documented. Therefore, we hypothesize that PCA can be an effective mycotoxin inhibitor and a novel substance to replace conventional mycotoxin binders. To warrant the above hypothesis, FB1 degradation was detected post treatment with PCA, or anti-FB1 monoclonal antibodies, or D-glucose, or silymarin at diverse temperatures and time points. *In vivo* study, SPF 11-day-old embryonated eggs were inoculated with the samples treated with PCA, or anti-FB1 monoclonal antibodies, or D-glucose, or silymarin. The hatching rate, body weight gain, and gastric lesions were monitored to assess detoxification.

## Materials and methods

### FB1 monoclonal antibody preparation

First, the conjugate FB (1)-BSA was inoculated into Balb/c mice, and one hybrid cell line 4G5 excreting monoclonal antibody against FB (1) was obtained by fusing mouse Sp2/0 myeloma cells with spleen cells from the immunized mice. Afterward, the hybridoma 4G5 was injected into the abdomen of Balb/c mice, and the anti-FB (1) mcAb was harvested from ascites, and the titer reached 6.4 × 10^4^ after purification with the caprylic/ammonium sulfate precipitation method. Finally, the cross-reactivity results displayed that the anti-FB1 monoclonal antibody was highly specific to FB1 and the affinity was amounted to 1.0 × 10^9^ L/M.

### Detoxification of PCA on FB1 *in vitro*

First, 1 ppm FB1 solutions (Pribolab Pte. Ltd., Singapore) were prepared as stock reagents prior to the experiment, and then 1 ml of FB1 was incubated with 100 μl of 1 mol/L of D-glucose (purity >97%, TCI Development Co., Ltd., Shanghai, China), PCA (purity >98%, TCI Development Co., Ltd., Shanghai, China), and silymarin (10% solution, Geriatric Hospital, Beijing, China), respectively. To assess detoxification, incubations were treated at 60, 70, and 80°C for 0.5, 1, and 2 h, respectively. Simultaneously, 1 ml of FB1 was cultivated with 100 μl of three dilutions (1:1,000, 1:2,000, and 1:3,000) of the anti-FB1 monoclonal antibodies (Lvdu Biotechnology Co., Ltd., Shandong, China) at 25°C and 37°C for 0.5, 1.0, and 2.0 h, respectively.

### Effect of detoxifications on growth and gastric ulcerations of the embryonated eggs

A total of 50 SPF 11-day old embryonated eggs were purchased from a commercial company (Boehringer Ingelheim Inc, Beijing, China) and randomly divided into 10 groups: four experimental groups (anti-FB1 monoclonal antibody group, D-glucose group, PCA group, and silymarin group) and six control groups (FB1, PBS, D-glucose, PCA, silymarin, and monoclonal antibody) (Table 1), with 5 chicken embryos per group. The experimental protocols were approved by an ethical reviewing board at China Agricultural University (approved code: IACUC20190912), based on guidelines from the Institutional Animal Care and Use Committee (IACUC). This follows humane protocols that minimize pain in the animals. All the hatching chickens were euthanized at the end of the study in a CO_2_ chamber using 100% CO_2_ at a flow rate of 10–30% of the chamber volume per minute, and the birds were observed for the absence of breathing activities and loss of heartbeat. The CO_2_ flow lasted for at least 1 min after breathing arrest. After confirmation of death, an additional secondary physical euthanasia was carried out before tissue collection as previously described (30).

**Table 1:** Experimental protocol of detoxification in embryonated eggs.

**Groups**	**Treatment**	**No. of embryonated eggs**
Monoclonal antibodies + FB1	23 μg FB_1_ + 100 μL 1:3,000 antibody	5
D-glucose + FB1	23 μg FB_1_ + 0.1 M 100 μL D-glucose	5
PCA + FB1	23 μg FB_1_ + 0.1 M 100 μL PCA	5
Silymarin + FB1	23 μg FB_1_ + 230 μg Silymarin	5
D-glucose control	0.1 M 100 μL	5
PCA control	0.1 M 100 μL	5
Silymarin control	230 μg	5
Monoclonal antibodies control	100 μL 1:3,000 antibody	5
FB1	23 μg FB_1_	5
PBS control	100 μL sterile PBS	5

All embryonated eggs were randomly divided into 10 groups, namely four experimental groups (the FB1 + monoclonal antibodies group, the FB1 + D-Glucose group, the FB1 + PCA group, the FB1 + Silmymarin group, five replicates per group) and six control groups (D-glucose control group, PCA control group, Silymarin control group, Monoclonal antibody control group, PBS control group and FB1 group, five chicken embryos per group). Before inoculation, poorly growing embryonated eggs were eliminated from the experiment.

Before inoculation, the median infective dose (EID_50_) of FB1-induced gastric ulceration in chickens was calculated to be 23.17 μg/ml using the Reed-Muench method as previously described (6). The samples were prepared according to the following protocols based on preliminary tests: 138 μg of FB1 were blended for 2 min and then 600 μl of 1 mol/L D-glucose, 600 μl of 1 mol/L PCA, and 600 μl of 1 mol/L silymarin were added. Afterward, the aforementioned three substances were incubated at 80°C for 2 h. As for the detoxification of anti-FB1 monoclonal antibodies, 138 μg of FB1 were pretreated with 600 μl of 1:3,000 antibodies and then incubated at 25°C for 2 h. The embryonated eggs were disinfected and then 100 μl of preparations per egg were inoculated into embryo albumens. Afterward, the embryonated eggs were incubated at 37°C for 21 days and monitored daily for mortality, development, and hatching numbers. Post hatching, both live birds and dead embryos were observed to determine the body weight gain and lesions of gastric ulceration in order to assess detoxification.

### FB1 detection

The samples were then cooled to room temperature before being tested for FB1 content using high-performance liquid chromatography (SPD-M20A, Shimadzu Corporation, Japan). Phenomenex prodigy ODS column (250 × 4.6 mm) was employed with a mobile phase of methanol:sodium biphosphate (77:23). The sample was detected at a flowing rate of 0.8 ml/min using the wavelength of 335 nm as previously described (6).

### Statistical analysis

The FB1 concentrations and lesion scores were statistically analyzed using SPSS 17.0 version to perform a one-way ANOVA with the LSD *post-hoc* test on at least three independent replicates. The *p*-values of <0.05 were considered statistically significant for each test, and when *p* < 0.01, the results were highly significant. The hatching rate was statistically analyzed using SPSS version 17.0 to perform a chi-square test with a categorical variable. A *p*-value of <0.05 was considered to be a significant difference for each test, and a *p*-value of <0.01 was considered to be highly significant difference.

## Results

### Detoxification of anti-FB1 monoclonal antibody, D-glucose, PCA, and silymarin

**Figure 1:**
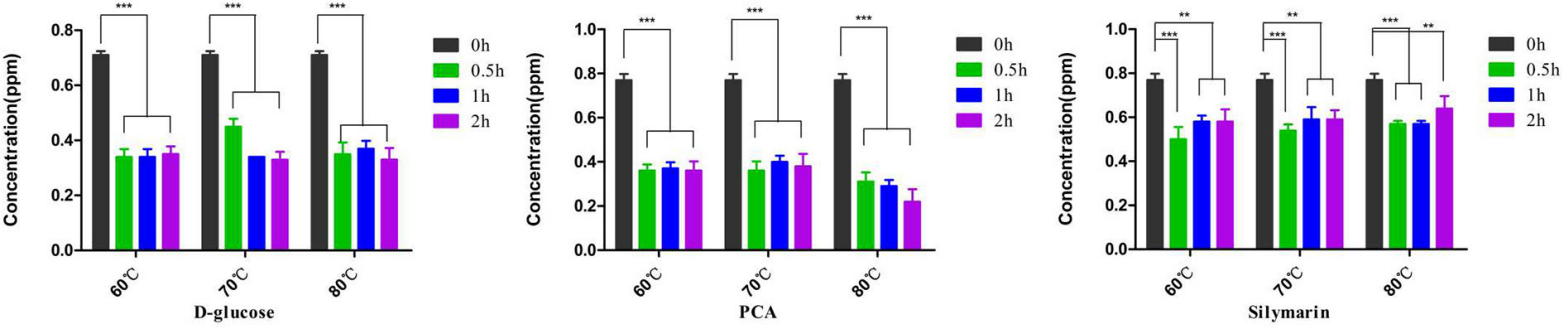
Detoxification of D-glucose, PCA, and silymarin post treatment with diverse temperatures and different durations. D-glucose and PCA possessed highly degradation activities on FB1, while silymarin possessed a detoxifying activity on FB1 degradation. **p < 0.01 and ***p < 0.001, D-glucose, PCA, and silymarin groups showed significant differences at 0.5–2 h compared to the initial treatment at 0 h. Regarding temperature on detoxification, the degrading effects of D-glucose and PCA were evident at 80°C for 2 h. However, no statistical difference was found in the silymarin-treated group from 60 to 80°C.

The FB1 preparations were incubated with D-glucose, or PCA, or silymarin at various temperatures and different time points (Figure 1). Regarding FB1 decontamination, 71.4% and 53.4% reductions were observed post treatment with PCA or D-glucose at 80°C for 2 h, which was comparable to 220 ppb and 330 ppb of FB1 residuals in the above two treatments. As for the optimal temperature, detoxification was inconsistent with temperature elevation and the maximal effect was determined to be at 80°C. As for time points, a time-dependent manner of the PCA treatment was observed at 80°C from 0.5 to 2 h, and FB1 removing concentrations were arranged from 59.2% to 71.4%. On the contrary, no significant difference (*p* > 0.05) of detoxification was found post treatment with D-glucose from 60 to 70°C for 2 h. Compared to PCA and D-glucose, a lower detoxifying effect on FB1 was observed in the silymarin-treated group compared to the initial treatment. For a prolonged time, no significant difference was found from 0.5 h to 2 h. The optimal detoxification was determined to be at 60°C for 0.5 h, and the concentrations were reduced from 770 ppb to 500 ppb, accounting for 35.1% degradation.

**Figure 2:**
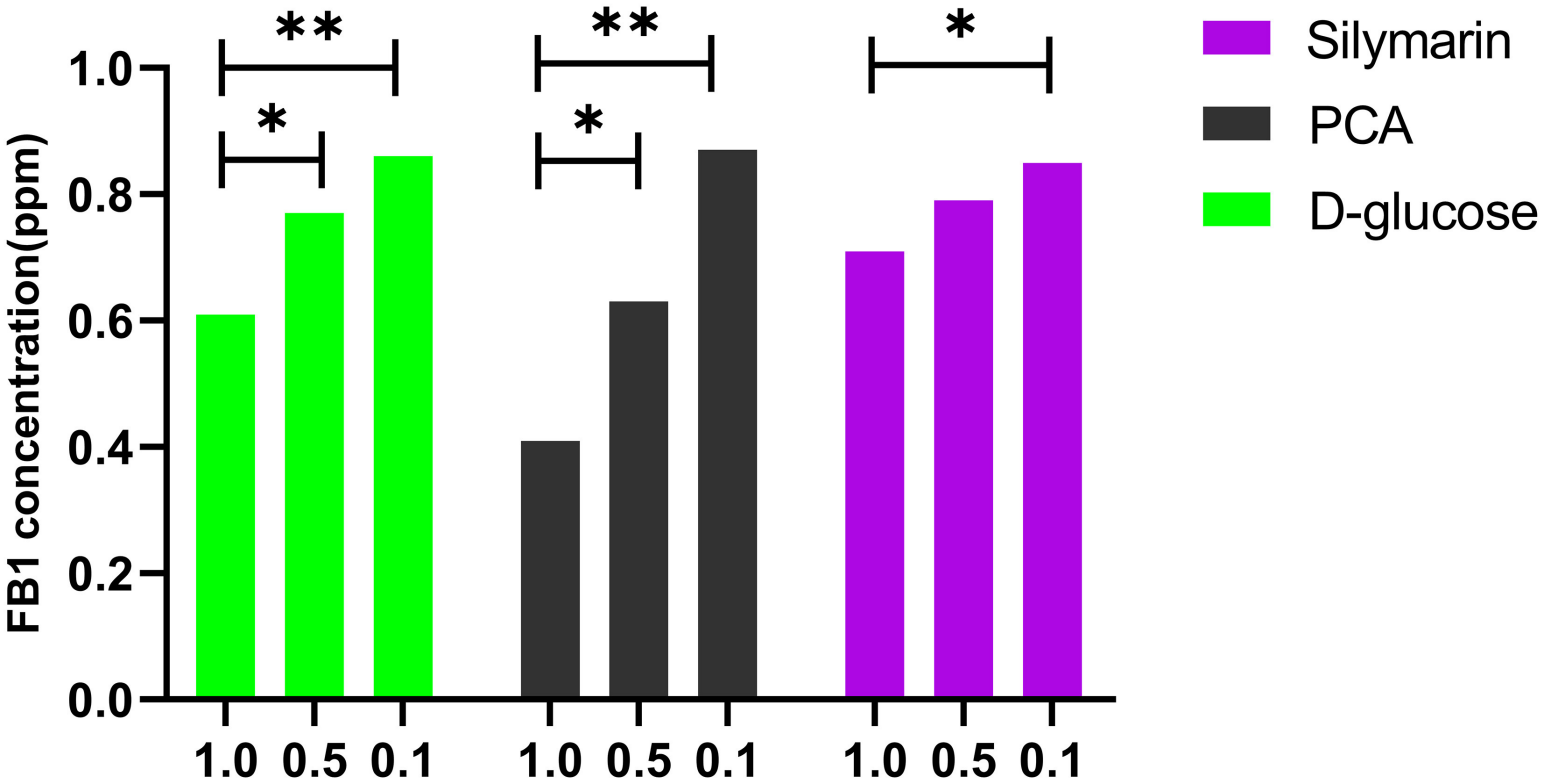
Effect of D-glucose, PCA, and silymarin on FB1 detoxification post treatment with different concentrations. The dose-dependent manners were detected in the D-glucose-treated group and PCA-treated group using HPLC. Significant detoxification of FB1 was found between 1.0 and 0.5 mol/L of concentrations in the above two groups at 80°C for 2 h, while a statistical difference was determined between 1.0 and 0.1 mol/L of the silymarin-treated groups at 60°C for 0.5 h (*p* < 0.05).

Subsequently, the dose-dependent manners were detected in the D glucose-treated group and PCA-treated group at 80°C for 2 h using HPLC. A significant difference was found between 1.0 and 0.5 mol/L concentrations (*p* < 0.05) in the above groups, while a statistical difference was determined between 1.0 and 0.1 mol/L of silymarin-treated groups at 60°C for 0.5 h (*p* < 0.05) (Figure 2).

**Figure 3:**
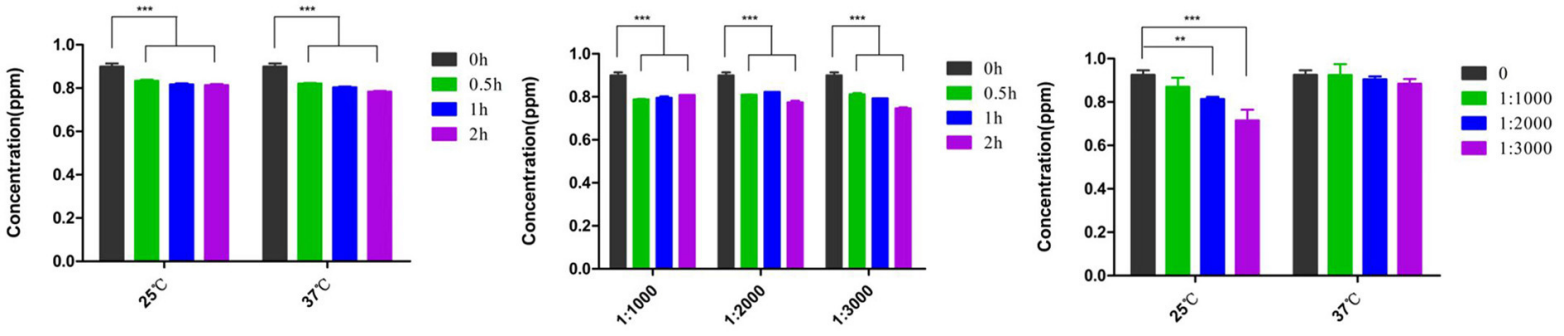
The detoxification of anti-FB1 monoclonal antibodies at different temperatures and time points. No significant difference in FB1 degradation was found between 25 and 37°C. A significantly decreasing trend was found in the 1:3,000 anti-FB1 monoclonal antibody-treated group at 25°C. Moreover, a reverse dose-dependent manner of detoxification was observed post treatment with diverse anti-FB1 monoclonal antibodies at 25°C. **p < 0.01 and ***p < 0.001.

The effects of the anti-FB1 monoclonal antibody on FB1 decontamination were assessed at different concentrations, temperatures, and time points (Figure 3). As for temperature treatment, no significant difference was found between 25 and 37°C from 0.5 to 2 h. However, FB1 degradations were associated with decreased anti-FB1 monoclonal antibody and prolonged time. No statistical difference (*p* > 0.05) was found between 1:1,000 dilution and 1:2,000 dilution from 0.5 to 2 h. Furthermore, significant detoxification was determined as treatment with a 1:3,000 concentration of anti-FB1 monoclonal antibody at 25°C for 2 h. The FB1 concentration was reduced to 0.689 ppm, and the degradation rate was 25.9% under optimal conditions. More interestingly, a reverse dose-dependent manner was observed post treatment with diverse anti-FB1 monoclonal antibodies at 25°C (Figure 3).

### Effect of detoxifications on growth and gizzard ulcerations of the embryonated eggs

**Figure 4:**
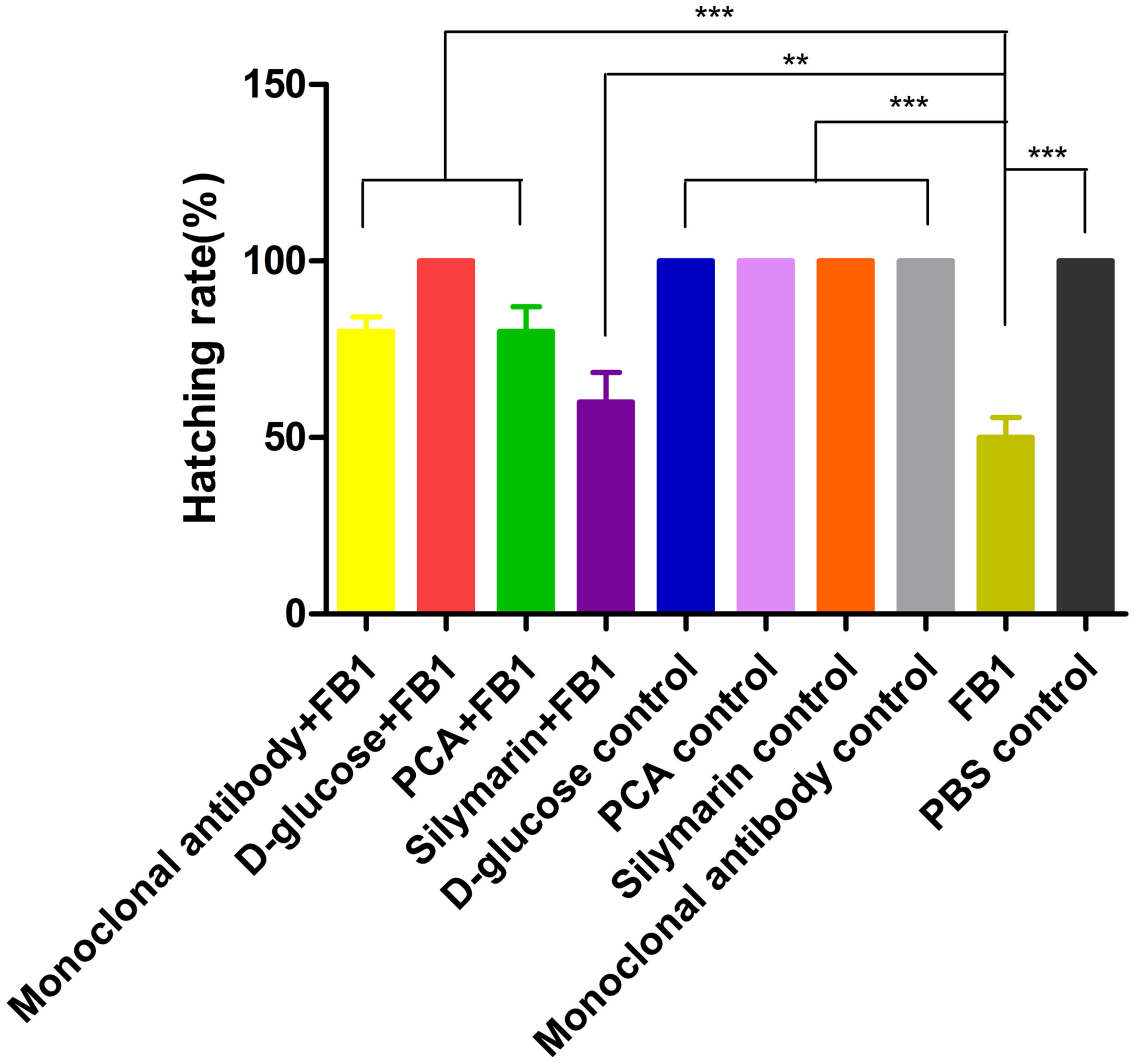
Hatching rate post FB1-treated with anti-FB1 monoclonal antibodies, or D-glucose, PCA, or silymarin. All control groups yielded 100%, while 80%, 70%, and 60% of the hatching rate were found in the PCA-treated group, anti-FB1 monoclonal antibody-treated group, and the silymarin-treated group, respectively. Compared to the FB1 group and the silymarin + FB1 group, a higher hatching rate was determined in the PCA-treated group and the anti-FB1 monoclonal antibody-treated group. **p < 0.01 and ***p < 0.001.

Upon breaking shells, a 100% survival rate was found in the D-glucose-treated group and other control groups except for the FB1 control group, while an 80% hatching rate was observed in the anti-FB1 monoclonal antibody-treated group and the PCA-treated group. However, the silymarin-treated group yielded 60% live chickens compared to 50% survival birds in the FB1 control group (Figure 4).

As for the body weight of the chickens, no significant difference was found among the D-glucose-treated group, the PCA-treated group, and the anti-FB1 monoclonal antibody-treated group, except for the FB1 group and the silymarin-treated group. Among them, the chickens in the silymarin group weighed only 33.1 g compared to 31.1 g of the FB1 group (Table 2).

**Table 2:** Body weight of chickens of different treated groups.

**Groups**	**Body weight (g)**	**Relative body**
		**weight gain%**
Monoclonal antibody + FB1	36.0 ± 0.14^a^	15.8^a^
D-glucose + FB1	35.90 ± 0.07^a^	15.4^a^
PCA + FB1	35.85 ± 0.07^a^	15.3^a^
Silymarin + FB1	33.11 ± 0.02^b^	6.5^b^
D-glucose control	35.9 ± 0.14^a^	15.4^a^
PCA control	36.0 ± 0.14^a^	15.8^a^
Silymarin control	35.85 ± 0.07^a^	15.3^a^
Monoclonal antibody control	35.80 ± 0.85^a^	15.1^a^
FB1	31.10 ± 0.07^b^	0.0
PBS control	36.74 ± 0.85^a^	18.1^a^

a Indicates *p* > 0.05 when compare with the average body weight of treatments and the control group in the same column.

b Indicates *p* < 0.05 when compare with the average body weight of treatments and control group in the same column. The data were expressed as the mean ± SD.

**Figure 5:**
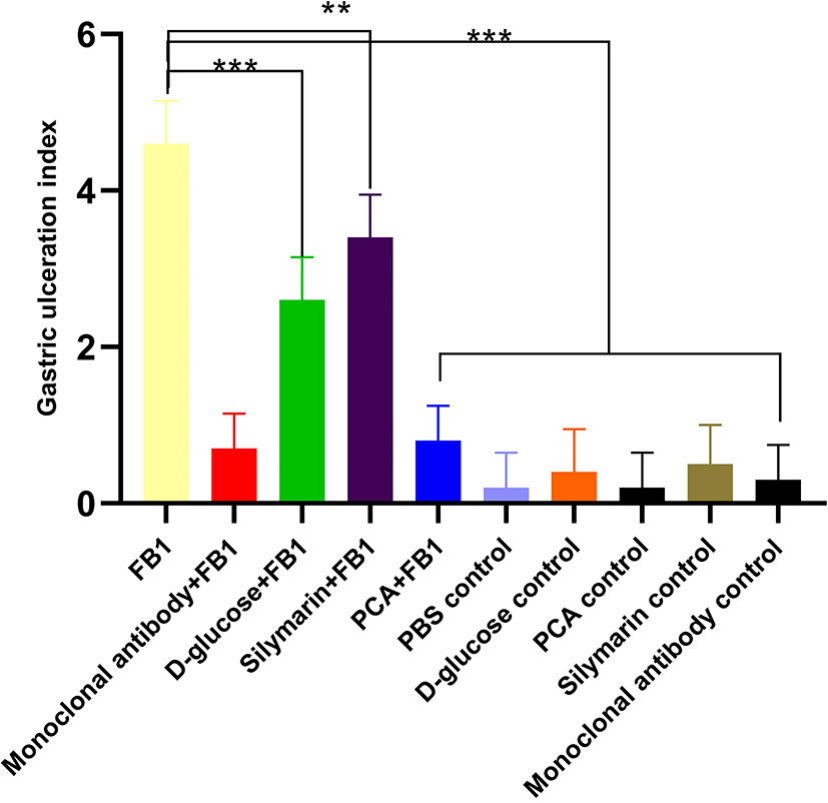
Gizzard ulceration index of chickens post inoculation with FB1 in different experimental groups. Postmortem, higher gizzard ulceration index was determined in the FB1 group, the D-glucose + FB1 group, and the silymarin + FB1 group compared to those of other groups. Compared to the D-glucose + FB1 group, both the PCA + FB1 group and the monoclonal antibody + FB1 group induced lower gizzard ulcerations (*p* < 0.01). The data were expressed as mean ± SD. **p < 0.01 and ***p < 0.001.

Postmortem, gastric lesion index was used to assess the detoxification post treatment. Obviously, the FB1-induced highly gizzard ulceration and gastric ulceration indexes were amounted to 4.4 ± 1.70, characterized as hemorrhagic inflammations and blood-staining lesions on the stomach. In terms of lesion index, lower lesions were determined in the PCA-treated group and the anti-FB1 monoclonal antibody-treated group compared to those of the D-glucose-treated group and the silymarin-treated group (*p* < 0.05). However, no significant difference (*p* > 0.05) was observed between the PCA-treated group and the anti-FB1 monoclonal antibody-treated group (Figure 5). However, gastric lesions were also observed in the D-glucose control group and the silymarin control group.

## Discussion

Our study focused on the most common fumonisin, FB1, in comparison to FB2 and FB2 (68:20:12) because of its various species-specific acute toxicological effects in domestic and laboratory animals as well as humans, having a significant effect on animal husbandry and posing a risk to human health (5). In this study, the detoxification of PCA on FB1 was investigated *in vitro* and *in vivo* with D-glucose, silymarin, PCA, and anti-FB1 monoclonal antibody. The purpose of the experiment was to develop more efficient mycotoxin detoxification for the poultry industry. The FB1 detoxing rate was amounted to 71.43 and 53.4%, respectively, when PCA and D-glucose were treated with FB1 at 80°C for 2 h. Additionally, silymarin and anti-FB1 monoclonal antibodies removed 35.15% and 25.9% of toxins. The dose-dependent detoxifications were determined in the PCA group and anti-FB1 monoclonal antibody. Furthermore, embryonated eggs were used to evaluate detoxification based on hatching rate, relative body weight gain, and gastric lesions. Although the D-glucose-treated group yielded a 100% hatching rate compared to 80% and 80% of hatching chickens in the PCA-treated group and anti-FB1 monoclonal antibody-treated group, lower gastric ulcerations were determined in the above two groups compared to the D-glucose-treated group and the silymarin-treated group. Therefore, PCA is a highly effective and safe FB1 detoxification in the poultry industry.

In our study, the PCA-treated group displayed significant detoxification *in vitro* and *in vivo*. However, the mechanism remains elusive. It is the first time that FB1 has been removed significantly post treatment with PCA. One main reason might be associated with antifungal activity. Mycotoxin usually exists in the field in foods and feeds the following infection of toxogenic fungus in the preharvest period, storage, and distribution of harvested products in the postharvest period. Three major factors influencing fungal development and mycotoxin production in foods are environmental conditions, agricultural practices, and susceptibility of the plant genotypes (1, 31). Recent reports showed that *Monotheca buxifolia* methanolic extract suppressed fungal biomass by 29–35% among the isolated compounds, and lupeol acetate and PCA resulted in 79–81% and 74–79% antifungal against *Macrophamina phaseolina*, respectively, which was very close to that of the fungicide mancozeb 83–84% (32). Second, PCA might reduce FB1 accumulation in the samples. In this study, the PCA-mediated FB1 detoxing rate amounted to 71.43% compared to the 25.9% detoxification of anti-FB1 monoclonal antibodies. In a previous study on mice, 9C11E6 monoclonal antibodies (mAb) against FB1 were generated from hybridoma cell lines, and the amounts causing 50% inhibition (IC50) of binding of 9C11E6 mAb by free FB1 were 20.1 ng/ml, with weak cross-reactivity, ~6.8% of FB1 not reacting with the hydrolyzed FB1 (33). Further mAB, H2 scFv was shown to have a 82-fold higher binding capacity compared to its parent mAb and could effectively bind to free FB1 with an IC50 of 0.11 μM and no cross-reactivity to deoxynivalenol, nivalenol, and aflatoxin was observed (34). PCA is a phenolic compound naturally occurring in various plant species. Phenolic acids have been frequently described for their contribution to defense to plant fungal pathogens, either through direct interference with the fungus or through the reinforcement of plant structural components acting as a mechanical barrier. Phenolic acids also are suspected of specifically reducing mycotoxin accumulation in plants because of their *in vitro* demonstrated ability to inhibit the production of various mycotoxins, including type B trichothecene mycotoxins and fumonisins. Among phenolic acids, derivatives of cinnamic acid, e.g., caffeic, ferulic, and *p*-coumaric acids, are the best recognized contributors to *Fusarium* head blight resistance. Furthermore, *Fusarium graminearum* was found to biotransform chlorogenic acid into caffeic, hydroxychlorogenic, and protocatechuic acids and caffeic acid into protocatechuic and hydroxycaffeic acids, contributing to counteracting fungal activities and its production of mycotoxins (21).

In the study, detoxifying FB1 is dependent on optimal temperature, and both D-glucose and PCA were found to detoxify FB1 contamination at 80°C for 2 h. In a previous report, FB1 in contaminated corn could precipitate the nonenzymatic browning reaction with 0.1 M D-glucose at 60 and 80°C, resulting in a 50% reduction at 80°C for 2 days vs. at 60°C for 8 days (35). FB1 and D-glucose can undergo the Maillard reaction to produce N-carboxymethyl-FB1, which is less toxic, and the cytotoxicity of the converted product to Vero cells was significantly lower than FB1 cytotoxicity (31, 36). In another investigation, FB-contaminated corn had a nonenzymatic browning reaction with D-glucose at 60 and 80°C, indicating that D-glucose could react with the free amine group of FB1 to achieve detoxification (35). Thus, FB1 might be eliminated from the contaminated foods by heating them in the presence of reducing sugar glucose. PCA, as a reducing acid, can react with the primary amino group of FBs in the structure of the hydroxyl and carboxyl groups and have a detoxifying effect similar to D-glucose. The test results further verified our hypothesis that PCA treatment may be used to detoxify FB1-contaminated feed.

Although chlorogenic acid, caffeic acid, ferulic acid, and vanillic acid were effective in reducing FB1 production, these phenolic acids were investigated *in vitro* studies and no evidence was reported to detoxify *in vivo* and applications in the poultry industry. In this experiment, 50% hatchability of chicken embryos was observed post inoculation with FB1, characterized by a lower body weight of chicken embryos and a higher gizzard ulceration index compared to the PCA-treated group and the anti-FB1 monoclonal antibody-treated group. This is the first time that gizzard ulcerations have been reduced greatly by PCA treatment, contributing to healthy baby chickens and a sustainable poultry industry. Recently, avian gizzard ulceration has been documented frequently in poultry industry, leading to consistent diarrhea, poor feed conversion, and poor economic beneficence. Feed-borne FB1 and DON were associated with poor hatchability and gizzard ulcerations in new-borne chickens using breeder eggs as a transportation vehicle (6). In routine feed additives, large mycotoxin binders are recommended for detoxification of contaminated cereal or feeds due to low cost. However, a clay-based bentonite binder failed to entirely alleviate the harmful effects of AFs or FBs, and no effective FB1 detoxification treatments are currently available (1, 16). Hence, it is recommended that mycotoxin binders be tested, particularly regarding animal safety and interactions with nutrients prior to their inclusion in diets (37). Recently, enzyme-based mycotoxin detoxification, including FBs esterase enzyme, has been shown to remove FBs and form less toxic compounds. Nevertheless, long incubation and detection times, high cost, and constant supply of enzymatic materials may be disadvantages for large-scale use (38).

Regarding FB1-induced immune suppression, PCA was documented to improve immunity and enhance antiviral activities (22, 23). It could be a noble strategy to do research on PCA products for the effective detoxification of FBs and other mycotoxins, which might be used as a feed additive in the commercial sector. Considering this application of the PCA molecule with low cost and large-scale production *via* fermentation technology for mycotoxin detoxification in human and animal food chains, thermal treatment with PCA might significantly reduce the free FB1 toxin level; nonetheless, the molecule may be transferred into a cryptic equivalent of unknown toxicity (39). To elucidate the implications of the observed decrease in FB1 concentration during heating, both the chemical structures and biological activities of FB1 degradation products must be clarified. Additional studies involving molecular studies and suitable bioassay approaches are intended to ascertain and chemically characterize FB1 reaction products.

## Conclusion

In conclusion, it is the first time that PCA-detoxifying FB1 has been fully evaluated both *in vitro* and *in vivo* trials. Based on the findings reported here, PCA is a promising detoxifying agent against FB1, reducing it up to 71.43% at 80°C for 2 h. Post treatment with FB1, PCA enhanced the hatching rate and healthy chickens by reducing gastric ulceration of progeny. Our data demonstrate that PCA is proposed as a novel substance for the detoxification of FB-contaminated grains and feeds. However, further research is required to optimize the concentrations that may make this product usable for commercial application with respect to the amount and kind of mycotoxin contamination.

## Data availability statement

All datasets generated for this study were included in the article/supplementary material, further inquiries can be directed to the corresponding author/s.

## Ethics statement

The animal study was reviewed and approved by Ethical Reviewing Board at China Agricultural University (Approved code: IACUC20190912).

## Author contributions

FW, YC, and YW: *in vitro* and *in vivo* study. MH and FW: original draft preparation. YW, HH, and XL: data preparation and analysis. MS and CH: conceptualization, editing, and funding acquisition. All authors have read and agreed to the final version of manuscript.

## Funding

This work was funded by Asian Regional Cooperation Fund (Grant no.12200111) and Higher Education Commission, Islamabad Research & Development Division (Grant no.14160).

## Conflict of interest

The authors declare that the research was conducted in the absence of any commercial or financial relationships that could be construed as a potential conflict of interest.

## Publisher's note

All claims expressed in this article are solely those of the authors and do not necessarily represent those of their affiliated organizations, or those of the publisher, the editors and the reviewers. Any product that may be evaluated in this article, or claim that may be made by its manufacturer, is not guaranteed or endorsed by the publisher.
